# Engineering Silk and Silk-like Proteins into High-Performance Fiber Materials

**DOI:** 10.3390/ijms27219455

**Published:** 2026-10-23

**Authors:** Chang Liu, Fuzhong Zhang

**Affiliations:** 1 Department of Energy, Environmental and Chemical Engineering, Washington University in St. Louis, Saint Louis, MO 63130, USA; cliu1@wustl.edu; 2 Division of Biological & Biomedical Sciences, Washington University in St. Louis, Saint Louis, MO 63130, USA; 3 Institute of Materials Science & Engineering, Washington University in St. Louis, Saint Louis, MO 63130, USA

**Keywords:** engineered silk proteins, silk-like protein fibers, molecular design, protein sequence engineering, mechanical performance, sustainable protein materials

## Abstract

Silk and silk-like proteins emerge as programmable building blocks for high-performance fiber materials because they can integrate mechanical robustness, biological compatibility, environmental responsiveness, and degradability within genetically defined polymers. Synthetic biology now enables precise control over protein sequence, repeat number, molecular weight, and modular architecture, creating opportunities to design fibers beyond naturally harvested silks. However, application-ready protein fibers require coordinated control across multiple length scales, from sequence-encoded motifs and molecular assembly to spinning-induced alignment, hierarchical network formation, and final property profiles. This review discusses recent advances in engineering silk and silk-like proteins into mechanically and functionally optimized fibers. We highlight how crystallite-forming motifs, amorphous domains, terminal domains, and folded or dynamic modules contribute to fiber strength, toughness, extensibility, damping, shape recovery, humidity stability, biodegradation, and recyclability. We further connect these sequence–structure–property relationships to applications in structural textiles, biomedical fibers, smart and protective systems, and sustainable materials. Finally, we discuss future opportunities in scalable recombinant production and data-driven sequence design. These advances are expected to accelerate the translation of engineered protein fibers into practical high-performance materials.

## 1. Introduction

High-performance protein fibers occupy a distinctive materials space because they combine outstanding load-bearing performance with biological and sustainability-related functions that are difficult to integrate in conventional synthetic polymers [1,2]. Silk and silk-like proteins are particularly compelling within this class. Silk proteins include silkworm fibroins and spider spidroins, together with their recombinant and engineered derivatives. Silk-like proteins here refer to engineered protein polymers designed to mimic or extend the sequence architectures, assembly behavior, or fiber properties of natural silks. Related fiber-forming systems based on non-silk proteins are also discussed to illustrate complementary molecular design strategies. Natural silks have long been valued for their rare balance of strength, toughness, extensibility, low density, biocompatibility, and degradability, motivating their exploration as fiber materials for technical, biomedical, and sustainable-polymer applications [3]. Protein engineering expands this rationale from using naturally harvested proteins to designing sequence-defined fiber-forming polymers. Through synthetic DNA design, amino-acid sequence, protein charge, repeat number, molecular weight, and modular architecture can be precisely controlled to fine-tune fiber properties. Meanwhile, microbial production offers a cost-effective, non-animal route toward potentially scalable manufacturing [1,2,3]. Thus, engineered silk and silk-like proteins provide a programmable platform in which mechanical performance, biological compatibility, degradation behavior, and application-specific functions can be integrated and tuned within the same material system.

Despite this promise, translating engineered silk and silk-like proteins into application-ready fibers often requires considering a combination of performance metrics. Load-bearing textiles and structural materials require combinations of strength, toughness, low density, extensibility, fatigue resistance, and dimensional stability, whereas biomedical and biointegrated fibers also require biocompatibility, controlled degradation, and processability; dynamic and protective fibers further demand damping, recovery, and stable cyclic response [3,4,5]. These properties are often interdependent, and their balance depends on protein architecture and fiber processing. For example, increasing the post-spin draw ratio can improve fiber strength and stiffness, but excessive drawing can reduce strain at break and toughness [6]. Hydration can further shift this balance by increasing amorphous-chain mobility and extensibility while reducing fiber strength, stiffness, and dimensional stability [7]. Further, practical translation is also constrained by the difficulty of producing high-molecular-weight repetitive proteins at high yield, maintaining concentrated protein dopes in a stable, soluble state, and controlling fiber properties under varying humidity and environmental conditions [2,6]. Therefore, a key challenge is to establish engineered protein–fiber platforms, in which mechanical performance, functional properties, processability, and degradability can be precisely controlled and balanced according to the requirements of specific applications.

**Figure 1:**
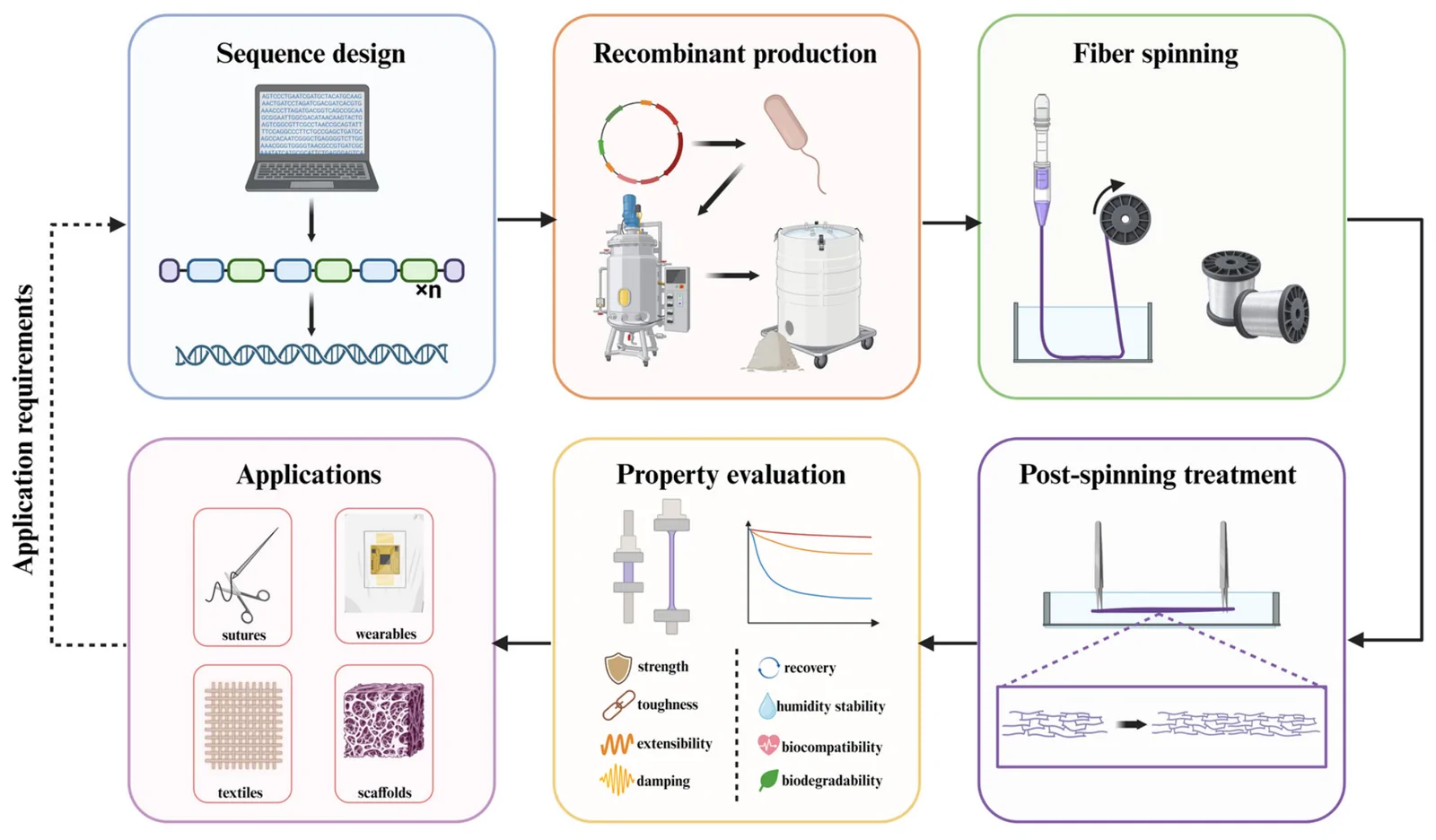
Engineering silk and silk-like proteins into high-performance fiber materials. Protein sequence designs are translated into fiber materials through recombinant production, spinning, and post-spinning processing. Fiber performance is evaluated against the requirements of intended applications, which in turn guide subsequent sequence design. This iterative design–build–test cycle connects molecular engineering, fiber fabrication, and application-specific performance targets. Created in BioRender. Liu, C. (2026). https://BioRender.com/k6x4guk.

In this review, we summarize recent advances in engineering silk and silk-like proteins into high-performance fiber materials, with an emphasis on how molecular design translates into hierarchical fiber structures and application-relevant properties (Figure 1). The discussion is organized around sequence–structure–property relationships that connect protein sequence features to fiber nanostructures, and macroscopic fiber mechanical or functional properties. By comparing representative fiber systems across different application contexts, we aim to reveal protein sequence design rules and processing choices that can be used to engineer protein fibers with application-ready property profiles.

## 2. From Protein Sequences to Fiber Structures and Properties

The mechanical properties of silk and silk-like protein fibers depend on both their amino acid sequences and the structures formed during fiber assembly. Sequence motifs provide the building blocks for ordered and flexible domains, whose interactions and connectivity govern force transfer and deformation. Spinning and post-spinning processing further shape protein packing and alignment, so fiber properties reflect the combined effects of molecular architecture and processing conditions.

### 2.1. Repeating Blocks as Structural Units for Mechanical Performance

Natural spider silk proteins contain hundreds of repeats with similar amino acid sequences. Each repetitive region contains a polyalanine and a glycine-rich motif. In silk fibers, the polyalanine motifs pack to form β-crystallites that bear load, while the glycine-rich motifs form amorphous domains around the β-crystallites, supporting chain mobility, extensibility, and energy dissipation. This sequence architecture allows silk proteins to be viewed as modular repeat polymers in which individual sequence blocks can be engineered to tailor silk nanostructures and their molecular interaction in the fiber form. For example, various amyloid-forming peptides have been used to replace the polyalanine motifs to form fibers with alternative β-nanocrystal sizes and higher crystallinity, leading to higher tensile strength and toughness [8,9].

Beyond these native-structural motifs, natural silk proteins also contain terminal domains, which undergo α-helix to β-sheet transition and dimerization during natural spinning, enabling end-to-end association in the fiber form. These terminal domains can be replaced with alternative self-interacting protein domains to promote interchain interactions without causing misfolding issues [10,11]. Additionally, functional blocks can be incorporated into silk proteins to introduce application-specific properties. Examples include fibronectin-derived motifs that promote cell adhesion [12], cysteine-containing tags that enable site-specific chemical functionalization [13], and fused enzyme domains that provide catalytic activity [14]. Thus, modular sequence design can tune mechanical properties such as tensile strength, Young’s modulus, extensibility, and toughness while introducing biological or chemical functions.

### 2.2. Hierarchical Assembly, Alignment, and Force Transfer in Silk Fiber Networks

**Figure 2:**
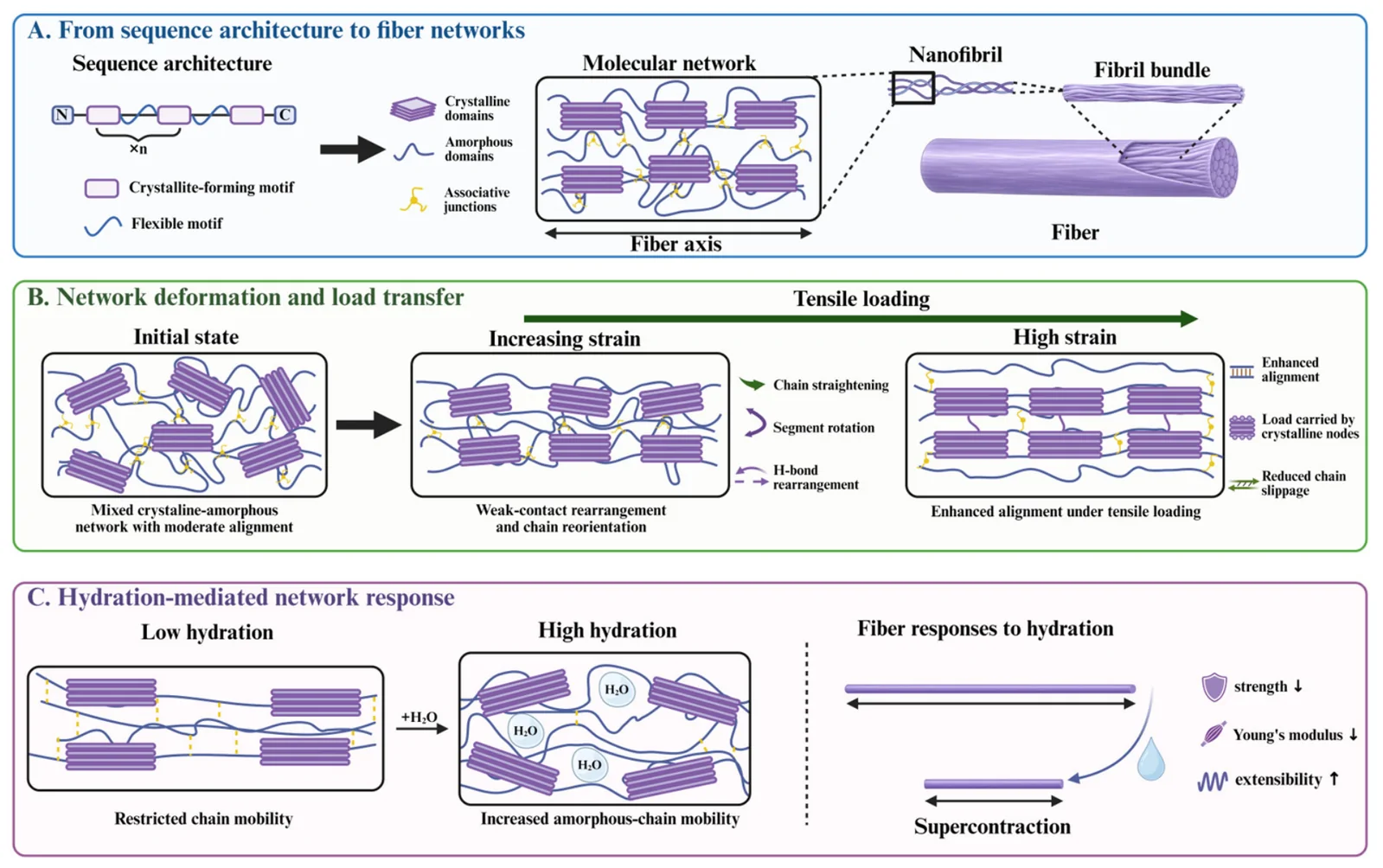
Sequence architecture, nanostructure, and network responses of silk and silk-like protein fibers. (**A**) Crystallite-forming and flexible sequence motifs contribute to interconnected molecular networks organized into macroscopic fibers. Nano-crystallites bridge different protein chains together, providing physical cross-links, while amorphous segments contribute to network connectivity and deformability. (**B**) Tensile loading promotes structural rearrangement, chain reorientation, and axial alignment. (**C**) Hydration responses lead to shorter fiber length, reduced tensile strength and Young’s modulus under high-humidity conditions. Upward and downward arrows indicate increases and decreases in the corresponding properties, respectively. Created in BioRender. Liu, C. (2026). https://BioRender.com/y6rbkhq.

In native spidroins, pH reduction promotes protein association through the N-terminal domain and helix-to-β sheet transition at the C-terminal domain, while flow stretches and aligns protein chains [10]. In native silkworm fibroin, flow-induced assembly is accompanied by reduced protein hydration and intermolecular β-sheet formation [15,16]. Recombinant spidroins carrying functional terminal domains can support pH-responsive assembly, whereas constructs lacking these domains rely on processing-induced association of repetitive regions [10]. Both native and engineered fibers can form interconnected networks of ordered nanocrystals and amorphous domains. β-Sheet-forming silk motifs and engineered amyloid sequences can form nanometer-scale crystallites through backbone hydrogen bonding and sequence-dependent side-chain packing [9,17]. Although a crystallite can contain β-strands from either a single protein chain or multiple chains, only those that bridge different protein chains function as effective intermolecular physical cross-links [18]. Meanwhile, glycine-rich and other flexible segments largely populate the amorphous matrix and connect the rigid crystalline domains. Interchain hydrogen bonds, van der Waals interactions, and, in some engineered proteins, electrostatic interactions provide additional connectivity within the amorphous domains. Interactions in the amorphous domains are generally weaker and less cooperatively organized than those within crystalline domains. Terminal-domain associations can provide additional intermolecular interactions. At larger length scales, these molecular components act together to create interconnected pathways for stress transfer from individual protein chains to the macroscopic fiber (Figure 2A) [19,20].

Under tensile loading, many silk and engineered silk-like protein fibers display an initial elastic deformation at low strain that is reversible upon unloading. This response primarily arises from reversible distortion of weak intermolecular interactions, such as hydrogen bonds and van der Waals contacts between protein chains, together with limited reorientation of β-sheet crystallites [18]. Above a characteristic threshold (i.e., the yield point), increasing numbers of weak interactions break, allowing greater mobility of the protein chains. With continued pulling, chains in the amorphous domains progressively straighten and align. For low-molecular-weight proteins or fibers with insufficient interchain crystallites, there are insufficient physical cross-links to restrict chain sliding. Deformation can then proceed with little increase in stress until fiber failure, producing the post-yield plateau observed in the stress–strain curves of many low-molecular-weight silk and silk-like protein fibers [9,18]. In comparison, high-molecular-weight recombinant silks and engineered amyloid–silk hybrid proteins can form more extensive networks of interchain crystallites. These crystallites are substantially stiffer than the amorphous domains and undergo relatively little deformation, enabling them to transfer load between protein chains throughout the fiber network. Further pulling leads to chain extension and alignment, local conformational transitions, and rearrangement of transient hydrogen bonds and other noncovalent interactions. These processes dissipate energy, while the progressive extension of chains produces strain stiffening, resulting in an upward curvature of the stress–strain curves (Figure 2B) [18,21,22].

Efficient force transfer further requires structural orientation and connectivity across multiple length scales. Axial alignment of both crystalline and amorphous domains promotes stress transfer along the fiber direction. While sequence architecture defines the spacing, dimensions, and interaction modes of these elements, spinning flow, coagulation kinetics, and post-spin drawing influence protein packing, orientation, and network connectivity within the fiber [7,11]. Hydration also modulates the network by increasing mobility within amorphous regions, which can induce softening or supercontraction while β-sheet crystallites remain as physical cross-links (Figure 2C) [7,23].

### 2.3. Fiber Spinning and Post-Spinning Processing as Structure-Forming Steps

Wet-spinning is currently widely used to spin recombinant and engineered silk and silk-like proteins. The process begins with a concentrated protein dope in various solvents, including aqueous buffer, hexafluoroisopropanol (HFIP), ionic liquids, formic acid, etc. Dope concentration, viscosity, solvent choice, and phase state determine whether protein chains can be extruded without premature aggregation while remaining sufficiently close to assemble [24]. In some systems, phase-separated, coacervate-like materials can provide a pre-organized state to facilitate shear-induced alignment and subsequent solidification [15,24,25].

The concentrated protein dope is then extruded through a spinneret into a coagulation or collection bath. During this step, shear force, solvent exchange, and in some cases pH or ionic changes, drive interchain association, β-crystallite formation, and fiber solidification [16,26]. Bath composition, residence time, protein concentration, flow rate, and reeling speed can potentially affect fiber diameter, morphology, β-sheet content, extensibility, strength, and water stability, although individual parameters may affect different subsets of these properties [26]. After initial solidification, post-spin drawing or stretching further imposes axial strain, increasing molecular alignment, crystallite orientation, and network uniformity. Post-spin drawing often improves tensile strength, Young’s modulus, and sometimes toughness, although excessive drawing can reduce extensibility or increase processing variability [26,27,28]. In addition to wet spinning, electrospinning provides a complementary route to nano- to submicron-scale fibers, nonwoven mats, and aligned fiber arrays. In this process, an electric field draws a charged protein solution into fine jets, and rapid solvent evaporation and subsequent deposition generate fibers with small diameters, high surface-area-to-volume ratios, and porous or interconnected architectures [29]. Overall, spinning and post-spinning parameters are not merely fabrication details; they are structure-forming variables that act together with molecular design to determine fiber performance.

## 3. Engineering Silk Protein Sequence for Enhanced Fiber Mechanical and Functional Performance

**Figure 3:**
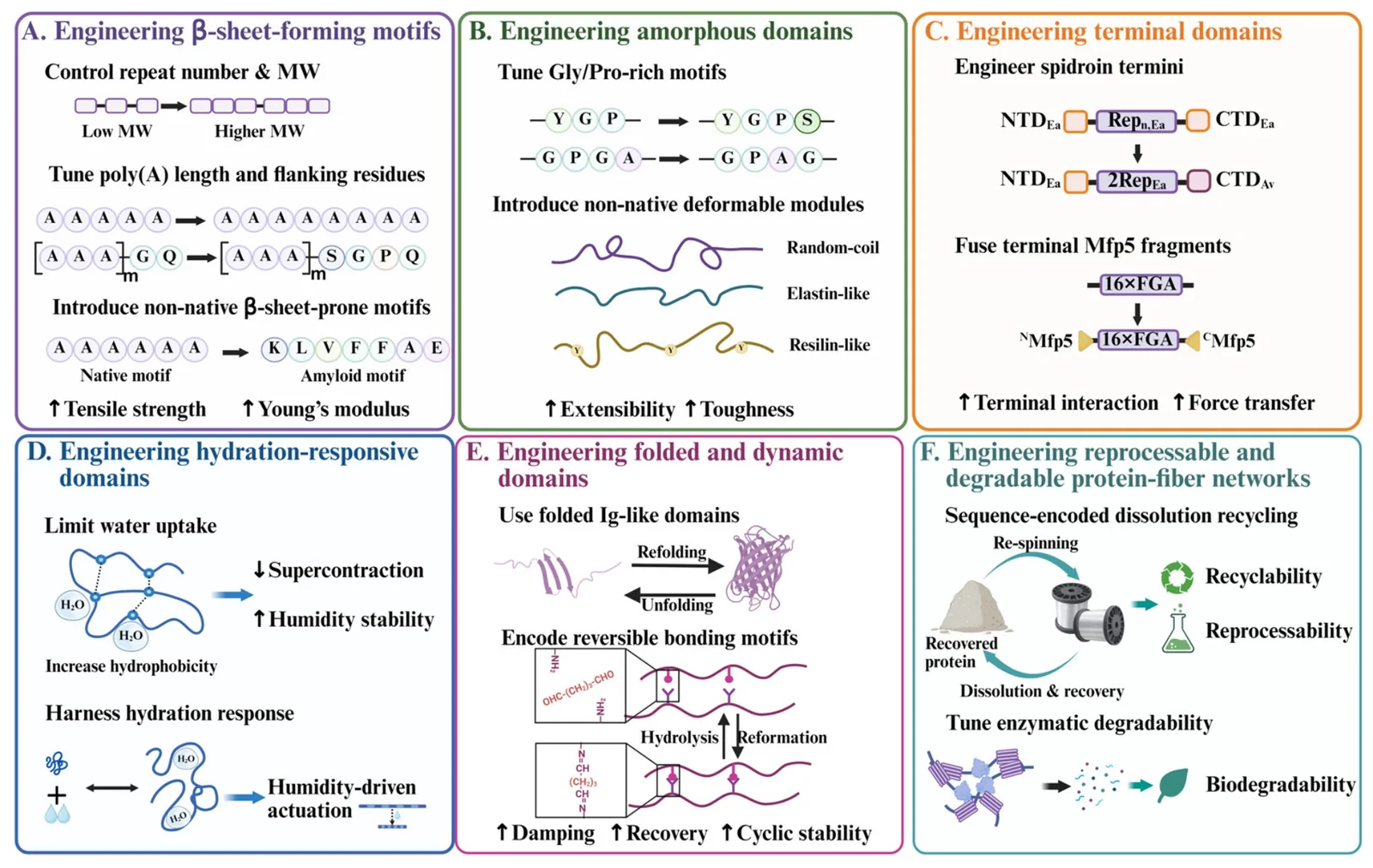
Sequence engineering strategies to modulate fiber mechanical properties or functional outcomes. (**A**) Engineering repeat numbers or β-sheet-forming domains to enhance fiber tensile strength and Young’s modulus. (**B**) Engineering amorphous domains to promote fiber extensibility and toughness. (**C**) Engineering protein terminal domains to regulate assembly and intermolecular association. (**D**) Engineering hydration-responsive sequence or domains to improve humidity stability or enable humidity-driven actuation. (**E**) Engineering folded domains or reversible bonding motifs to enhance energy damping, shape recovery, and performance during cyclic force loading and unloading. (**F**) Engineering protein–fiber networks to enable recycling and biodegradation. Upward and downward arrows beside property labels indicate increases and decreases, respectively. Created in BioRender. Liu, C. (2026). https://BioRender.com/u8cv13u.

**Figure 4:**
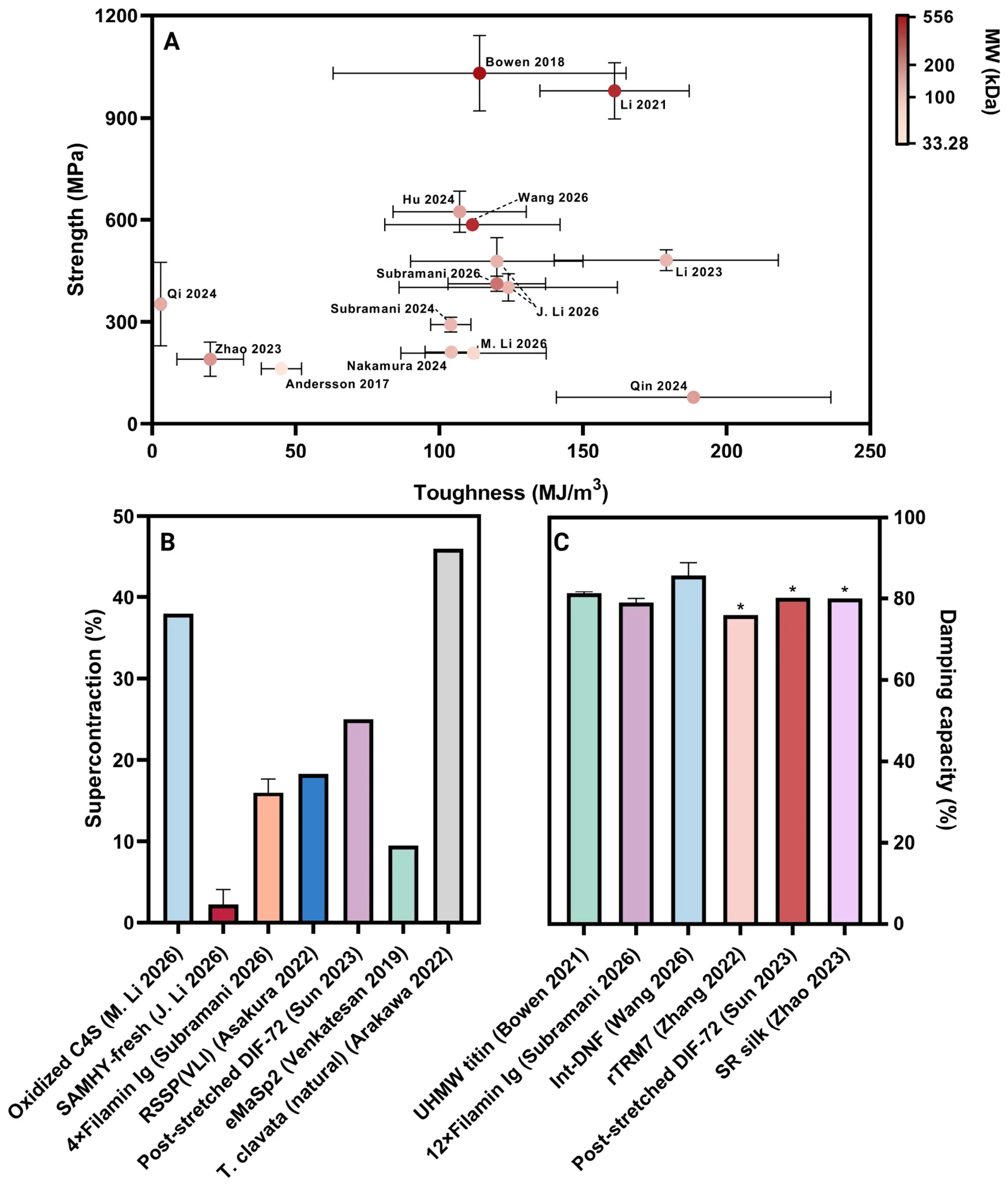
Mechanical and functional properties of representative silk and silk-like protein fibers. (**A**) Comparing tensile strength and toughness of silk and silk-like protein fibers from various engineering strategies. Selected fibers represent the best mechanical performance within each engineering strategy. Marker color indicates protein molecular weight (kDa). Horizontal and vertical error bars indicate the reported variability in toughness and tensile strength, respectively. (**B**) Supercontraction of representative fibers. (**C**) Damping capacity of representative fibers. Data sources: (**A**) [8,9,11,30,31,32,33,34,35,36,37,38,39,40]; (**B**) [32,38,39,41,42,43,44]; (**C**) [5,34,39,40,44,45]. In (**B**,**C**), testing conditions varied among studies. Error bars indicate available standard deviations, reported numerically or estimated from published figures. Asterisks (*) indicate values calculated from published loading–unloading data or curves.

Protein sequence engineering provides a direct route to translate the structural principles described above into tunable fiber performance. Recent studies have used engineered sequence motifs and modular protein domains to control fiber crystallinity, amorphous-domain deformability, intermolecular interaction, hydration response, dynamic recovery, and lifecycle-related functions (Figure 3). These strategies are grouped by their primary engineering targets, although individual strategies sometimes affect multiple fiber properties. Table 1 summarizes the mechanical and functional outcomes reported for representative examples of each strategy. Figure 4A presents fibers with the highest tensile strength or toughness achieved through each engineering strategy as summarized in Table 1. These two mechanical properties provide complementary measures of a fiber’s load-bearing and energy absorption capacities and are the most widely used metrics for evaluating fiber mechanical performance. Figure 4B,C present supercontraction and damping capacity of fibers cited in this review, respectively.

### 3.1. Increasing the Number of Repetitive Domains

It has long been established and extensively demonstrated that increasing protein molecular weight (MW) can enhance fiber strength and modulus [9,30]. High-MW proteins contain more repetitive domains and therefore have a greater capacity to form intermolecular interactions, particularly β-sheet crystallites assembled from multiple protein chains. These crystallites act as strong physical cross-links that facilitate force transmission throughout the molecular network and limit chain slippage [18]. As a result, high-MW protein fibers exhibit higher modulus, more pronounced strain hardening, and ultimately greater tensile strength.

However, high-MW, highly repetitive proteins are notoriously difficult to express in heterologous hosts because of challenges associated with genetic instability, mRNA degradation, biased codon usage, and imbalances in tRNA availability [2]. To solve this problem, circular mRNAs can be used to improve the stability of repetitive transcripts and increase protein yields in microbial hosts [46]. Furthermore, split-intein-mediated ligation and polymerization have enabled the assembly of short protein precursors into high-MW protein chains [30,47]. Using split-intein-mediated ligation, a recombinant spider silk protein containing up to 192 repeating units of polyalanine and glycine-rich regions has been synthesized. This protein has a MW of 556 kDa, and its fiber exhibits an ultimate strength of 1.03 ± 0.11 GPa, a modulus of 13.7 ± 3.0 GPa, an extensibility of 18 ± 6%, and a toughness of 114 ± 51 MJ/m^3^, fully replicating the mechanical properties of natural *Trichonephila clavipes* dragline silk fibers [30]. Additionally, using spontaneous in vivo protein polymerization, repetitive titin proteins with molecular weights as high as 2 MDa have been expressed [5]. Several repetitive proteins were also produced at high titers, reaching up to 8 g/L [11]. In the silkworm *Bombyx mori*, repeat sequences from the major ampullate spidroin 1 (MaSp1) have been fused to the fibroin light chain for expression and incorporation into cocoon fibers. Extending the fibroin light-chain promoter from approximately 1 to 4 kb was found to improve expression, although increasing the number of MaSp1 repeats from 8 to 32 reduced expression under the same promoter [48]. These advances have substantially expanded the capacity to manufacture engineered proteins for high-performance material applications.

### 3.2. Engineering β-Sheet-Forming Motifs to Control Crystalline Reinforcement

Because the crystallite-forming β-sheet motifs are responsible for load-bearing in the fiber form, they can be engineered to increase fiber tensile strength and Young’s modulus. Recent efforts in tuning the length, spacing, interaction, and folding of β-sheet motifs via protein sequence engineering have led to controlled crystalline-domain formation and fiber mechanics (Figure 3A).

Extensive sequencing studies have revealed that different spidroins contain polyalanine repeats of varying lengths, typically ranging from 5 to 14 residues depending on the spider species and spidroin type. Altering the length of the polyalanine motif is expected to change the dimensions and stability of β-sheet crystallites, thereby affecting fiber load-bearing behavior. When MaSp1 was heterologously expressed and spun by the silkworm *Bombyx mori*, increasing the length of its polyalanine segment from 8 to 16 alanine residues increased the Young’s modulus and ultimate tensile strength by 22% and 13%, respectively [48]. In contrast, when recombinant silk proteins were expressed in bacteria and processed by artificial wet spinning, increasing the polyalanine length from 5 to 12 residues produced a nonmonotonic effect on modulus and strength, with the eight-alanine variant exhibiting the highest values. This eight-alanine variant also formed robust nanofibrillar assemblies, which may facilitate the development of intermolecular β-sheet crystallites during artificial spinning [31]. These findings indicate that polyalanine length influences not only β-sheet length but also fiber assembly, thereby affecting mechanical performance through multiple mechanisms.

The residues flanking polyalanine motifs have also been proposed to influence β-sheet length and stability, consequently affecting fiber mechanics [41,49]. Another strategy is to introduce cysteine residues at both ends of the polyalanine segment [32]. Following spinning-induced alignment of the polyalanine regions, oxidative treatment promotes disulfide-bond formation between cysteine residues, substantially increasing fiber tensile strength. Thus, engineering the amino acids flanking polyalanine motifs provides another means of modulating β-crystalline reinforcement and tuning the mechanical properties of recombinant silk fibers.

Additionally, the entire polyalanine motif can be replaced with alternative β-sheet-forming peptides to promote β-crystallite formation [9,50]. From a protein-structural perspective, polyalanine is not necessarily the most effective sequence for driving β-sheet assembly. Consequently, fibers wet-spun from low-MW recombinant spider silk proteins are often largely amorphous because artificial spinning alone is insufficient to induce extensive β-crystallization [28]. In contrast, steric-zipper-forming peptides derived from amyloid proteins are well known for their strong propensity to assemble into stable β-sheet structures. Replacing polyalanine with these zipper-forming peptides produces hybrid amyloid–silk proteins that form fibers with substantially greater β-sheet crystallinity—approximately 4–5-fold higher than that of comparable recombinant silk fibers [9,51]. Because these peptides contain bulkier side chains, the inter-sheet spacing in amyloid–silk fibers is approximately 0.91 nm, a value characteristic of amyloid structures and nearly twice that observed in natural spider silk. In addition, the stronger interstrand interactions within amyloid-derived β-sheets confer high stability on nanofibrils [51]. Together, these structural features lead to marked improvements in fiber mechanics, with modulus and ultimate tensile strength reaching up to 2.0- and 3.3-fold higher, respectively, than those of recombinant silk fibers of similar molecular weight. Subsequent blending of amyloid–silk proteins with different sequences provides an additional means of tuning fiber strength and modulus by varying the proportion and identity of the β-sheet-forming motifs [8]. Moreover, replacing repetitive polyalanine sequences with more sequence-diverse amyloid motifs reduces overall sequence similarity and facilitates microbial expression. This strategy enabled the production of amyloid–silk proteins containing 128 repeating units, whose fibers exhibited gigapascal-level tensile strength and toughness exceeding 160 MJ m^−3^, surpassing those of some natural spider silks [9].

### 3.3. Engineering Amorphous Domains to Control Fiber Stretchability and Energy Dissipation

The glycine-rich amorphous domains contribute to chain mobility, deformability, and reversible structural rearrangement under tensile stress [52]. These domains can be engineered to control fiber breaking strain, toughness, and energy dissipation (Figure 3B).

The Spider Silkome Database provides extensive sequence information on conserved motifs within the amorphous domains of spidroins, including GGX, GXG, GPQ, and QQG motifs [41]. Studies using low-MW recombinant silk variants have shown that modifying these motifs can substantially alter fiber extensibility and toughness [33]. In MaSp2, for example, substitution of YGP with YGPS or QQG with SQG increased both ultimate strain and toughness by 38–96%, without significantly affecting tensile strength or modulus. In the YGPS variant, the YGP-to-YGPS substitution was accompanied by a GPGA-to-GPAG substitution (Figure 3B) [33]. In contrast, replacing QGPY with QGPSGY in the amorphous domain reduced breaking strain and toughness by 20–30%, while producing only small increases in ultimate tensile strength and modulus [33]. These findings suggest that sequence modifications within flexible amorphous motifs can strongly influence fiber stretchability and energy absorption, likely by altering local chain mobility and intermolecular interactions. However, it remains unclear whether these sequence–property relationships are broadly applicable to other types of spidroins.

Another strategy for engineering the amorphous domain is to introduce non-native deformable modules. Elastin-like protein fibers provide a good example: through silk-to-elastin ratio control and elastin-like block design, researchers can tune thermal responsiveness, swelling, deformability, and mechanical behavior [53,54]. Similarly, incorporation of resilin into transgenic silk improved silk strength and resilience, showing that intrinsically disordered elastic proteins can be used as flexible mechanical modules [34]. Spidroin-mimetic fibers containing β-sheet-forming modules together with a non-spidroin random-coil module achieved high toughness, illustrating how engineered amorphous segments can preserve deformability while crystalline modules maintain network integrity [35].

Additionally, nonrepetitive spacer domains provide another native-derived strategy: incorporating a globular amyloidogenic MiSp spacer domain from *Araneus ventricosus* into chimeric spidroins facilitates self-assembly, improves molecular homogeneity, and enhances fiber strength [36].

### 3.4. Engineering Terminal Domains to Control Protein–Protein Interactions

Beyond engineering the crystalline and amorphous domains, terminal-domain interactions provide another route to improve fiber mechanics. During natural spinning, the pH decreases from approximately neutral in the silk gland, where the spidroin dope is stored, to acidic near the end of the spinning duct. The N-terminal domains undergo pH-dependent dimerization, whereas acidification and shear destabilize the α-helical C-terminal domains and promote conformational rearrangements associated with β-sheet-rich assembly [10,19,55]. Recent studies of native dragline silk have further identified Arg–Tyr cation–π interactions as sequence-encoded molecular stickers that promote phase separation and connect protein condensation with β-sheet assembly [56]. Together, these findings motivate engineering strategies that incorporate interacting terminal domains (Figure 3C).

A particularly successful chimeric minispidroin, NT2RepCT, was engineered by combining the N-terminal domain and two repetitive units from *Euprosthenops australis* MaSp1 with the C-terminal domain from *Araneus ventricosus* MiSp1 [37]. These terminal domains confer high aqueous solubility and pH-responsive assembly, enabling the protein to be concentrated and spun entirely from aqueous solution. This combination of terminal domains from different spidroins demonstrates the modularity of the approach and its potential for scalable recombinant silk production using aqueous spinning. However, extending this architecture to substantially longer repetitive regions presents challenges in achieving concentrated dopes while preserving correctly folded terminal domains. These constraints have so far limited the production of high-strength artificial silk fibers through aqueous spinning.

An alternative strategy for introducing terminal association is to fuse split mussel foot protein 5 (Mfp) fragments to both termini of a repetitive silk or amyloid–silk protein. Mfp is intrinsically disordered, and its fragments interact through multiple cation–π, π–π, and hydrophobic interactions without requiring a specific folded interface or precise orientation [57,58]. Fusion of split Mfp fragments to both protein termini promotes end-to-end intermolecular association, enhances the axial alignment of β-sheet motifs, and substantially increases fiber strength and toughness [11]. The amino acid composition of these Mfp-derived terminal domains can be further engineered to tune fiber hydration and supercontraction. For example, in the engineered silk–amyloid–mussel foot protein SAM_HY_, all Lys and Arg residues in the C-terminal Mfp fragment were replaced with His, and all Ser residues were replaced with Tyr. These substitutions increased the aromatic and hydrophobic character of the terminal domain, thereby limiting water uptake. SAM_HY_ fibers exhibited only 2.2 ± 1.9% supercontraction at >90% relative humidity, while retaining high tensile strength and toughness through the combined contributions of Tyr-rich aromatic interactions and axial alignment of β-nanocrystals [38]. These studies establish Mfp-derived termini as sequence-tunable interaction modules that enhance effective chain connectivity, β-nanocrystal alignment, and mechanical robustness without requiring proteins of very high molecular weight.

### 3.5. Engineering Protein Hydrophobicity to Tune Supercontraction and Moisture-Triggered Functions

Natural spider silks and many engineered protein fibers exhibit strong hydration responsiveness. Under high humidity or upon exposure to water, water molecules infiltrate the amorphous domains and disrupt hydrogen bonds, causing substantial fiber shrinkage and loss of tensile strength, a phenomenon often called supercontraction [38,39]. Although these structural and mechanical changes are generally undesirable in textile and structural applications, they can be harnessed for specialized functions such as humidity sensing, shape-memory behavior, and actuation [32,42]. Sequence-engineering strategies that either suppress supercontraction or convert water uptake into controlled contraction are therefore highly valuable (Figure 3D).

One strategy for suppressing supercontraction is to limit water ingress and reduce hydration-sensitive interactions within non-crystalline domains. To address the pronounced supercontraction of ADF-3 silk fibers, a recombinant protein termed RSSP(VLI) was engineered by replacing all QQ dipeptides with VL and individual Q residues with I, thereby increasing protein hydrophobicity. Supercontraction, calculated as the relative decrease in fiber length upon hydration, was reduced from 23% in fibers made from the unmodified protein to 18% in RSSP(VLI) fibers [43]. More recently, engineering the hydrophobicity of the C-terminal Mfp5 domain of SAM proteins reduced fiber supercontraction from approximately 40% for fibers containing the native Mfp5 sequence to 2.2% for SAM_HY_ fibers [38]. This modular design allows supercontraction to be tuned through the hydrophobicity of the C-terminal Mfp5 domain while fiber mechanical properties are largely controlled by the amyloid–silk repeat region. Additionally, proteins containing folded immunoglobulin (Ig)-like domains offer another route to moisture resistance. Due to their high hydrophobicity, filamin-derived Ig fibers exhibited low supercontraction (16% at 99% relative humidity) and retained most of their tensile strength (89%) at high humidity. Meanwhile, the hydrogen-bonding networks associated with their Ig folds provided high strength and toughness [39].

Conversely, hydration responsiveness can be harnessed as a functional output. Genetically engineered MaSp2-derived fibers containing polyalanine and proline-rich motifs have been used to achieve humidity-responsive shape-memory behavior, converting supercontraction-like contraction and recovery into programmable actuation [42]. Targeted motif substitution in MaSp2-based artificial spider silk has further shown that sequence motifs can be screened and exchanged to tune tensile properties, structural orientation, and hydration response [33]. Recent recombinant spidroin designs have also introduced cysteine-mediated reinforcement near β-crystalline domains to preserve β-sheet alignment under high humidity while enabling strong, reversible humidity-driven actuation [32]. Representative supercontraction values for selected fibers are summarized in Figure 4B. Effective engineering of hydration-responsive domains therefore involves tuning hydrophobicity, hydrogen-bonding networks, interchain cross-links, and folded-domain stability to produce either moisture-stable structural fibers or fibers that deliberately convert water uptake into motion or shape recovery.

### 3.6. Engineering Folded and Dynamic Domains to Program Damping, Recovery, and Cyclic Stability

Dynamic and protective fibers require more than high strength and toughness; they must dissipate mechanical energy, recover after deformation, and maintain performance under repeated loading. Folded and dynamic domains provide sequence-level modules for these functions because they can undergo reversible unfolding/refolding, transient interaction rupture and reformation, or humidity-assisted network recovery during loading–unloading cycles (Figure 3E).

A major strategy is to use mechanically responsive folded protein domains as sacrificial mechanical units. Titin-based fibers established this design principle by using tandem immunoglobulin-like domains as repeat modules. High-molecular-weight titin polymers produced in microbial hosts formed fibers with high strength, toughness, damping capacity, and humidity-assisted recovery, suggesting that aligned Ig-like domains can form load-bearing interchain junctions that resist chain slippage while permitting domain unfolding under high strain to dissipate energy [5]. More recent Ig-domain fibers broadened this approach by comparing sequence-diverse domains from titin, myomesin, and filamin [39]. In this system, Ig domain hydrophobicity correlated with fiber assembly and tensile strength, folding energy correlated with damping capacity, and hydrogen-bonding networks influenced humidity-induced weakening. High-molecular-weight 12 × Filamin Ig fibers further combined high tensile strength, toughness, damping capacity, and nearly complete shape recovery over repeated cycles, illustrating how domain identity and repeat number can be tuned separately to program load-bearing and dynamic recovery [39].

A complementary strategy is to encode reversible bonding sites within deformable protein networks. Dynamic imine fiber chemistry uses lysine-rich elastin-like repeats as reactive modules, in which lysine residues provide reversible imine crosslinking and the random-coil elastin-like backbone provides chain mobility, enabling strong and tough protein fibers with recoverability after fatigue or acid-induced weakening and humidity-triggered actuation [44]. Dual-dynamic-network protein fibers extend this principle by combining a squid-ring-teeth-derived β-sheet-forming module, a resilin-inspired elastic module, and a lysine-rich elastin-like crosslinking module, integrating hydrogen bonding, dynamic imine crosslinks, and entropy-driven elasticity to achieve high damping during cyclic loading [40]. Related byssus-inspired protein fibers further show that hydration-dependent hydrogen bonding, disulfide bonds, and Ca^2+^-carboxyl coordination can coordinate wet-state extensibility with strength and self-recovery [45]. Representative damping capacities of selected fibers are summarized in Figure 4C. Overall, folded-domain engineering, dynamic-network engineering, and reversible sacrificial deformation could allow protein fibers to dissipate energy, recover shape, and retain mechanical function over repeated loading cycles.

### 3.7. Engineering Reprocessable and Degradable Protein–Fiber Networks for Sustainable Lifecycles

Recyclability, reprocessability, and biodegradation are emerging design targets for engineered silk and silk-like protein fibers. Conventional polymer end-of-life strategies, including mechanical recycling, chemical recycling, and solvent-based dissolution–precipitation, are generally applied to post-use or post-industrial waste streams and can be limited by property degradation, energy demand, or material-compatibility constraints [59,60,61]. Engineered protein fibers offer an additional design lever because recyclability, reprocessability, and biodegradation can be influenced at the design stage by controlling crystallinity, chain cohesion, solvent accessibility, dynamic interactions, and protease-accessible domains. Earlier silk regeneration studies showed that dissolution, regeneration, and re-spinning can recover useful silk materials and fibers, but regenerated fibers often display deteriorated mechanical performance due to reduced molecular integrity and processing-induced structural loss [62]. Recent engineered protein fibers extend this concept by treating recyclability as a sequence-encoded material function (Figure 3F).

A representative strategy is dissolution recycling of silk–amyloid–mussel proteins. In SAM_HY_ fibers, the silk–amyloid repeat core forms load-bearing β-nanocrystals, whereas engineered Mfp-derived terminal domains affect hydrophobicity, solvent accessibility, and intermolecular interactions. Lys/Arg-to-His and Ser-to-Tyr substitutions in the C-terminal Mfp fragment reduced formylation-prone residues while preserving π–π interactions and β-sheet alignment, allowing fibers to dissolve rapidly in aqueous formic acid solution and be re-spun over multiple cycles with retained strength, toughness, low supercontraction, and structural integrity [38].

Biodegradation is governed by fiber structure and protein sequence features. Enzymatic degradation studies of recombinant spider silk eADF4(C16) showed that material format affects proteolysis: particles, films, and nonwoven meshes made from the same protein degrade at different rates, and chemical crosslinking reduces both degradation rate and extent [63]. For silkworm silk materials, degradation depends on enzyme type, molecular weight, β-sheet crystallinity, amorphous-region accessibility, processing history, porosity, and morphology [64]. Recent strategies further tune degradation by embedding silk-degrading enzymes inside silk devices, shifting degradation from surface erosion toward internally initiated bulk degradation [65]. Conversely, crosslinking crystalline and amorphous regions can increase silk fibroin resistance to enzymatic degradation, improving stability but reducing degradability [66].

Effective lifecycle-oriented fiber engineering therefore balances stable load-bearing interactions with reversible or biologically accessible network features [67]. Sequence designs that combine tunable crystallinity, dynamic or solvent-disruptable intermolecular interactions, and protease-accessible amorphous domains may allow protein fibers to retain performance during use while remaining recyclable, reprocessable, or degradable at end of life.

**Table 1:** Representative sequence-engineering strategies and their resulting mechanical or functional properties of silk and silk-like protein fibers.

Engineering Strategies	Protein Domains	Resulting Mechanical or Functional Properties	Examples	References
Increase repeat number and molecular weight	Repeat architecture	Enhanced strength, strain, and toughness	FGAILSS (16 to 128 repeats): tensile strength increased from 230 ± 34 to 980 ± 80 MPa [9].	[9,30,68]
Optimize polyA length	β-sheet-forming domains	Enhanced tensile strength and modulus	N15C-8A: 2.2-fold tensile strength relative to N16C-5A [31].	[31,48]
Changing polyA flanking residues	β-sheet-forming domains	Enhanced tensile strength and modulus	Oxidized C4S: 2.7-fold tensile strength relative to cysteine-free Ori4 [32].	[32,33]
Replace polyA with strong β-sheet-forming peptides	β-sheet-forming domains	Enhanced strength, modulus, and toughness	(A_3_I)_3_-A_14_: toughness of 146 MJ/m^3^, versus 18 MJ/m^3^ for NT2RepCT [50].	[9,50]
Tune Gly/Pro-rich silk motifs	Amorphous domains	Enhanced strain and sometimes toughness	GGX-only fibers: 2.9-fold extensibility relative to GGX/GPGGX fibers [52].	[33,52]
Add ELP repeats	Amorphous domains	Enhanced strain and toughness	As-spun AQK5-CTD: toughness of 196.6 ± 42.2 MJ/m^3^ [35].	[35,53,54]
Add resilin domain	Amorphous domains	Enhanced tensile strength and resilience. Reduced Young’s modulus	SR silk: 20.5% higher tensile recovery than WT silk [34].	[34]
Blend different proteins	Composite fiber network	Tunable strength, strain, modulus, and toughness	75:25 16xFGA/16xGDV blend: 47–55% higher toughness than either pure component [8].	[8]
Engineer terminal domains	Terminal domains	Enable aqueous spinning	NT2RepCT: aqueous spinning at a dope concentration of 500 mg/mL [37].	[37]
Use Mfp as terminal domains	Terminal domains	Enhanced strength, strain hardening, and toughness. Reduced supercontraction	Bi-terminal Mfp5 fusion doubled 16xFGA-fiber toughness to 118 ± 21 MJ/m^3^ [11].	[11,38]
Add spacer domains	Amorphous domains	Enhanced fiber formation, integrity, and strength	MiSp spacer addition: tensile strength increased from 124 ± 89 to 352 ± 123 MPa [36].	[36]
Increase protein hydrophobicity	Hydration-sensitive domains	Reduced supercontraction and enhanced humidity stability	SAM_HY_: supercontraction of 2.2% at 94% relative humidity [38].	[38,39,43]
Use reversible interacting motifs	Dynamic modules	Increased damping, recovery, and cyclic stability	UHMW titin: damping capacity of 81% at 30% strain [5].	[5,34,39,69]
Use dynamic bonds	Dynamic modules	Self-recovery, fatigue recovery, and damping	DNF-12: damping capacity of 89% after 10 cycles (20% strain; humidity-mediated recovery) [40].	[40,45]
Design fiber recycling	Recyclable fiber networks	Recyclability and retained re-spinning performance	SAM_HY_: tensile strength of 478 ± 69 MPa after three recycling cycles [38].	[38,62]

## 4. Application-Matched Fiber Design

Recombinant silk or engineered protein fibers have numerous potential applications, including load-bearing textiles, structural fibers, surgical sutures, drug delivery vehicles, tissue scaffolds, wearable sensors, smart textiles, and many others. Different applications require distinct combinations of mechanical, physical, biological, and functional properties as well as environmental response, degradability, and lifecycle consequences. To meet these application-specific requirements, proteins must be carefully designed to balance all these properties while remaining compatible with manufacturing processes.

### 4.1. Fibers for Load-Bearing Applications

Fibers with high modulus, strength, and toughness can be used as load-bearing textile and structural fibers, including ropes, cables, protective fabrics, and reinforcement layers in aerospace, automotive, sports, marine and other high-performance-demanding systems. Current high-performance fibers mainly include carbon, aramid, and UHMWPE fibers. They offer high strength, high modulus, and low density; some also provide chemical, thermal, and fatigue resistance, dimensional stability, and durability [70,71,72]. Protective fabrics and ballistic composites further require high energy absorption, impact resistance, and stable performance under a wide range of environmental conditions [73]. Engineered protein fibers have achieved gigapascal-level tensile strength and can exceed some conventional high-performance fibers in toughness [5,9]. However, their relatively low initial modulus and yield strength, together with insufficient resistance to high-rate impact, remain important limitations for applications such as protective fabrics and ballistic composites. Future protein–fiber engineering should therefore focus on enhancing initial modulus and yield strength while preserving high toughness and energy-dissipation capacity, thereby expanding their potential in demanding structural and protective applications.

### 4.2. Fibers for Biomedical Applications

Fibers for biomedical applications require a different property profile. Surgical sutures must possess sufficient tensile strength to maintain wound closure for a desirable amount of time, reliable knot security, suitable handling characteristics, minimal tissue reaction, predictable strength retention, and reliable absorption kinetics over clinically relevant timescales [74,75]. Meanwhile, scaffolds for tissue engineering need suitable 3D architectures, and mechanical properties tailored to the specific tissue being regenerated [76,77]. Current sutures are commonly produced from natural materials such as silk and collagen, as well as absorbable synthetic polymers, whereas fibrous tissue-engineering scaffolds frequently use collagen, silk fibroin, and biodegradable polyesters [75,77]. Engineered protein fiber materials are particularly attractive for biomedical applications as they can combine favorable mechanical properties with biocompatibility and programmable absorbability [76,77]. For example, fibronectin-functionalized recombinant spider silk has been assembled in the presence of mammalian cells into 3D networks of extracellular-matrix-like microfibers that supported cell proliferation and differentiation while exhibiting wet-state mechanical properties similar to arterial walls [12]. Future engineering should emphasize precise control over degradation kinetics enabling fiber strength, structural persistence, and resorption to be matched to the requirements of specific tissues and stages of healing [76,77].

### 4.3. Fibers for Dynamic and Smart Textile Applications

Fibers for dynamic and smart textile applications include wearable sensors for human-motion monitoring, textile-based soft actuators and artificial muscles for shape or porosity control, and impact-adaptive protective textiles [78,79]. Demonstrated applications include conductive–elastomer composite yarns for sensing, twisted or coiled nylon and polyethylene yarns for actuation, and shear-thickening-fluid-impregnated aramid fabrics for impact-responsive protection [78,79]. Applications involving repeated deformation or impact require controlled energy damping, time-dependent recovery, and fatigue resistance during cyclic loading [39,40,44]. Wearable sensors impose additional requirements, including flexibility, stretchability, breathability, skin compatibility, signal stability, safety, washability, and durability under repeated bending or stretching [80,81,82]. Protein fibers are attractive for these applications because sequence-level design and reversible intermolecular interactions can combine load bearing, energy dissipation, recovery, and environmental responsiveness within a single fibrous material [39,44]. For example, engineered MaSp2 fibers showed humidity-sensitive shape memory and high recovery stress, while cysteine-reinforced recombinant spidroin fibers enabled strong and reversible humidity-driven torsional actuation [32,42]. Together, these examples position protein fibers as candidate platforms for soft actuators, adaptive textiles, wearable interfaces, and responsive protective materials. Further engineering should prioritize stable and repeatable responses under variable humidity and temperature, long-term fatigue and wash resistance, and scalable integration into textile manufacturing.

### 4.4. Fibers for Other Applications

Beyond the above application classes, high-performance protein fibers and nanofibrils can also be used in filtration membranes, functional coatings, resource-recovery systems, and recyclable fibrous materials. For example, recombinant spider-silk nanofiber nonwoven meshes have been explored for PM2.5 filtration, where application performance is governed by fiber diameter, mat porosity, specific surface area, air permeability, filtration efficiency, and network-level mechanical integrity [29]. A silk–elastin-like protein fused with lanthanide-binding peptides has been used for selective rare-earth-element recovery [83]. Furthermore, protein-based fibers can be used in recyclable textiles to reduce the need for virgin fiber production and lower energy use and carbon emissions [84]. Such applications align well with recyclability and biodegradability of protein fibers [38,84]. The representative applications discussed in this section illustrate how distinct mechanical and functional requirements can guide protein sequence engineering.

## 5. Future Opportunities for Predictive and Scalable Fiber Design

Future progress in engineered silk and silk-like protein fibers will depend on connecting molecular design with scalable production, reproducible processing, and predictive property control. A persistent challenge is that the sequence features that improve mechanics often complicate manufacturing. High molecular weight, long repetitive genes, and β-sheet-prone segments can increase chain overlap, entanglement, and load transfer, but they also create problems in gene stability, translation burden, protein aggregation, solubility, purification, and concentrated dope preparation [2,85]. Commercial translation also requires cost-effective fermentation, downstream recovery, dope formulation, solvent management, and fiber spinning, rather than high performance under laboratory-scale conditions alone [67,85]. Solvent-free thermomechanical fusion of native silk fibers also highlights the value of hierarchy-preserving processing routes that avoid solvent-intensive regeneration [86]. Promising routes include more stable repetitive-gene construction, split-intein-based protein polymerization, secretion-based production, high-cell-density production, and engineered proteins with precisely controlled fiber properties [2,47,87]. These strategies should be evaluated not only by protein titer and purity, but also by cost per mass of usable fiber, batch-to-batch reproducibility, processing compatibility, environmental footprint, and retained mechanical performance after scale-up [67,85].

Another frontier is predictive design. Earlier modeling-guided work on recombinant silk-like block copolymers showed that block composition, chain length, and shear-flow-induced network organization can jointly govern fiber formation and mechanical performance [88]. A large-scale spider-silkome dataset now provides a sequence–property foundation by linking spidroin sequence diversity with measured dragline-silk mechanical, structural, thermal, and hydration-related properties [41]. Recent deep-learning frameworks have begun linking spidroin repetitive-region sequences to fiber-level mechanical properties and identifying influential motifs and candidate mutations associated with strength, toughness, and related mechanical outputs [89]. Recent work further combined generated and natural sequences with structure prediction and steered molecular dynamics to evaluate sequence-dependent unfolding behavior and nanomechanical properties [90]. More broadly, AI tools are shifting protein engineering from structure prediction toward inverse sequence design. AlphaFold provides a sequence-to-structure foundation [91], while ProteinMPNN and RFdiffusion support backbone-conditioned sequence design and de novo protein generation [92,93]. ForceGen extends this trajectory to mechanics-conditioned sequence generation from nonlinear unfolding responses [94]. However, predictions of protein structure or molecular unfolding behavior alone do not establish the performance of spun fibers, which also depends on intermolecular assembly and processing history [88,89,90]. A practical priority is therefore to build datasets linking sequence, processing conditions, and measured fiber properties, and to validate model-designed proteins through fiber fabrication and testing under defined conditions. Artificial-silk roadmaps already emphasize iterative design–simulation–production–spinning cycles, while automated biomateriomics platforms show how robotic fabrication and machine-learning-guided experiment selection can close the loop in materials discovery [95]. Adapting this logic to protein fibers could shift the field from empirical optimization toward predictive, application-specific, and manufacturable material design.

## 6. Conclusions

Engineered silk and silk-like proteins have moved from bioinspired mimics toward programmable fiber-forming polymers in which sequence, molecular weight, modular architecture, processing, and hierarchical assembly can be deliberately coordinated. Current studies have clarified how β-sheet-forming motifs, amorphous domains, terminal domains, and folded or dynamic modules contribute to distinct mechanical and functional outputs. These insights provide a growing design toolkit for constructing fibers with application-matched property profiles. The next stage is to convert this toolkit into standardized and predictive platforms: scalable production, integrated sequence–structure–property datasets, and data-driven design workflows should allow target properties to guide protein sequence engineering and fiber processing. Achieving this transition will be essential for translating engineered protein fibers into practical structural, biomedical, smart, and sustainable materials.

## Data Availability

No new experimental data were generated. All data analyzed are available in the cited publications.
